# To the Editors of the Medical and Physical Journal

**Published:** 1802-03

**Authors:** 

**Affiliations:** Norfolk


### *To the Editors of the Medical and Phyſical Journal,*

GENTLEMEN,

IN the Science of Medicine there has ever been, and perhaps ever muſt be, much difference of opinion; yet, as by the colliſion of facts, ſparks are often produced to light us in our ſearch after truth, a *temperate Controverſy* may generally be productive of good. But the iſſue of *invective* is widely different; it gives birth to tortuous aſſertions, to perſonal reflections unworthy the man of ſcience, and engages a place in the mind which would be much better occupied by the ſubject in diſpute than by any reference to the contending parties.

I have been an attentive reader of your Journal from its commencement; and although I have, in common with others, obſerved it contained ſeveral things deſerving of cenſure, yet does it afford much to commend. I could not, therefore, peruſe with indifference the *illiterate* and *illiberal* perſonalities which have lately appeared in it. I am altogether unacquainted with any one of the parties; I attack not the *men*, but the *ſentiments* they have publiſhed. I am *inimical* to perſonal reflections in whatever manner they are publiſhed; but I *deteſt*, and will ever laſh, the production which teems with *unprovoked* obloquy; — nor can I altogether exclude the Editors from a ſhare of the blame. They will, perhaps, urge in reply, that the *Name* of the writer fixes all the evil on himſelf; or, though they publiſh ſome things anonymouſly, they are in poſſeſſion of the Author's real name. This anſwer does not ſatisfy me; *invective* on all occaſions, and all *perſonalities* whatever, ſhould be baniſhed from your pages. You have the power, and you ought to exerciſe it. Such prunings may offend a few irritable Correſpondents, but that is far better than to diſguſt the moſt reſpectable of your Readers by the admiſſion of ſcurrilities. I would wiſh to propoſe the following Queries to your Readers, if you deem them conſiſtent with the above ſentiments.

1. Has Philo-Medicus been refuſed literary honours, or does he conſider it as a neceſſary proof of his *literary candour*, that he ſhould ſcatter his obloquy upon *every* Member of an Univerſity, whatever may have been the order of his admiſſion to its honours, or however amply he may have been qualified to receive them in other approved literary ſeminaries?

2. Does it conſiſt with the dignity, duty, or honeſty of a Phyſician, to preſcribe in Hieroglyphicks, the key to which is in the excluſive poſſeſſion of one man, and conſequently unintelligible to every other member of the Profeſſion? *

3. Is

* We do not know what this Query alludes to. EDIT.

3. Is not your Communication from Abingdon,† a palpable breach of that *modesty in controversy* which it would seem inclined to inculcate, when it charges a Physician with *impetuosity*, *pride*, and *confidence*, because he acts upon principles which *he* believes to be derived from good and sufficient premises?

4. Is a Physician, or any other duly qualified Medical Character, to be charged with arrogance,‡ because he dares to think for himself, although *all* he so dares to think may not be correct?

5. Should not the animadversions of every Medical Controvertist be directed to the *subject matter* only, and without any regard being had to the *Authors* of opinions in dispute?

6. If criticisms be admitted as to the *manner* rather than to the *matter* of philosophic communications, in what Number of the Journal will some of its Correspondents criticise themselves?

I am, &c.

*Norfolk*, 6 *Feb.* 1802. ARISTARCHUS, JUN.§

§ We can assure A. that a numerous class of our readers are so far from agreeing in sentiment with him, that they say, "If you reject the salt of controversy, your Ragout will soon become as insipid as those of your predecessors."

